# Mechanical Properties of Medical Microbubbles and Echogenic Liposomes—A Review

**DOI:** 10.3390/mi16050588

**Published:** 2025-05-17

**Authors:** Hussain Alsadiq, Zahra Alhay

**Affiliations:** 1 Department of Mechanical Engineering, University of Prince Mugrin, Medinah 42241, Saudi Arabia; 2 Health Sciences Center, University of Oklahoma, Oklahoma City, OK 73104, USA; zahra-alhay@ouhsc.edu

**Keywords:** microbubbles, echogenic liposomes, ultrasonic contrast agents, Rayleigh–Plesset equation, acoustofluidics, shell elasticity

## Abstract

Lipid-shelled microbubbles (MBs) and echogenic liposomes (ELIPs) have been proposed as acoustofluidic theranostic agents after having been proven to be efficient in diagnostics as ultrasonic contrast agents. Their mechanical properties—such as shell stiffness, friction, and resonance frequency—are critical to their performance, stability, oscillatory dynamics, and response to sonication. A precise characterization of these properties is essential for optimizing their biomedical applications, however the current methods vary significantly in their sensitivity and accuracy. This review examines the experimental and theoretical methodologies used to quantify the mechanical properties of MBs and ELIPs, discusses how each approach estimates shell stiffness and friction, and outlines the strengths and limitations inherent to each technique. Additionally, the effects of parameters such as temperature and lipid composition on MB and ELIP mechanical behavior are examined. Four characterization methods are analyzed, including frequency-dependent attenuation, optical observation, atomic force microscopy (AFM), and laser scattering, their advantages and limitations are critically assessed. Additionally, the factors that influence the mechanical properties of the MBs and ELIPs, such as temperature and lipid composition, are examined. Frequency-dependent attenuation was shown to provide reliable shell elasticity estimates but is influenced by nonlinear oscillations, AFM confirms that microbubble stiffness is size-dependent with smaller bubbles exhibiting higher shell stiffness, and theoretical models such as modified Rayleigh–Plesset equations increasingly incorporate viscoelastic shell properties to improve prediction accuracy. However, many of these models still assume radial symmetry and neglect inter-bubble interactions, which can lead to inaccurate elasticity values when applied to dense suspensions. In such cases, using modified frameworks like the Sarkar model, which incorporates damping and surface tension explicitly, may provide more reliable estimates under nonlinear conditions. Additionally, lipid composition and temperature significantly affect shell mechanics, with higher temperatures generally reducing stiffness. On the other hand, inconsistencies in experimental protocols hinder direct comparison across studies, highlighting the need for standardized characterization methods and improved computational modeling.

## 1. Introduction

Lipid-shelled microbubbles (MBs) and echogenic liposomes (ELIPs) have been proposed as acoustofluidic theranostic agents after being proven efficient as ultrasonic contrast agents in diagnostic applications. These agents consist of gas-filled microbubbles encapsulated by a lipid shell, which provide stability and allows for targeted delivery. These carriers, with sizes that range from several microns to submicrons, can be utilized to encapsulate a wide range of therapeutic agents, including both hydrophilic and hydrophobic compounds, and deliver them to targeted sites within the body. The acoustic impedance difference between the gas core and surrounding fluid (which constitutes most human tissue) makes these agents echogenic, allowing real-time traceability.

To fully understand and optimize the performance of lipid-shelled microbubbles or ultrasonic contrast agents, it is essential to characterize their mechanical properties. This involves studying the response of the microbubbles to various mechanical forces, such as ultrasound waves or external pressure. The shell thickness and elasticity of microbubbles significantly impact their volumetric oscillation and cavitation, which are crucial for enhancing their drug delivery and therapeutic efficacy [1].

The investigation of the shell parameters of the delivery agent is a prerequisite to studying their acoustofluidic behavior, as shell parameters are crucial inputs in models used to predict the microbubbles’ translation velocity arising from sonication [2]. Characterizing the mechanical properties of lipid-shelled microbubbles or ultrasonic contrast agents, helps understanding their stability, elasticity, and resonance frequencies. This information is crucial for fine-tuning the design and functionality of these agents, as well as for ensuring their safety and effectiveness in both diagnostic and therapeutic applications. The mechanical properties of MBs and ELIPs, such as shell stiffness and resonance frequencies, are not only important for their stability and response to acoustic waves but are also directly tied to their clinical efficacy. For instance, in diagnostic ultrasound imaging, the enhanced contrast provided by MBs depends heavily on their ability to oscillate under acoustic pressure. Furthermore, in drug delivery applications, ELIPs must be able to withstand external pressures while remaining stable long enough to reach target sites. Therefore, optimizing these mechanical properties is essential for improving the precision and efficiency of ultrasound-based therapies.

The mechanical properties of MBs and ELIPs are influenced by the properties of their surrounding fluid, such as viscosity and specific weight. As a result, it is impossible to assess their mechanical properties without accounting for the fluid’s influence. To illustrate, the following is considered; if the encapsulated gas pressure in MB is considered in mechanical and electrical circuits analogies, as spring and capacitor, respectively, the viscosity of the surrounding fluid will be represented as friction in the mechanical analogy and electrical resistance in the electrical analogy [3].

**Figure 1:**
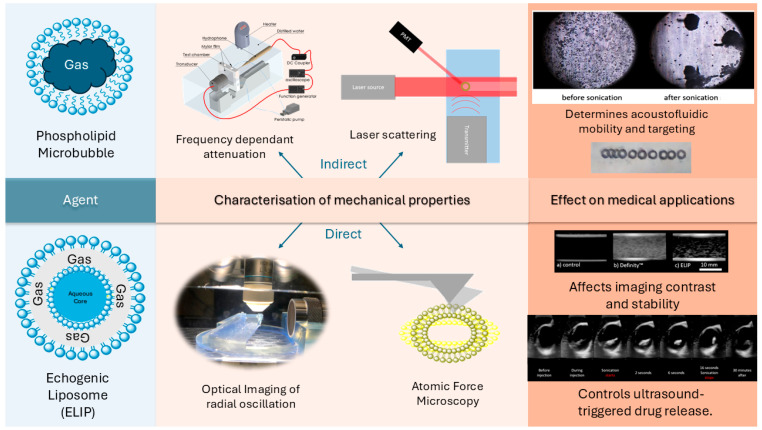
Conceptual overview of microbubble and echogenic liposome (ELIP) characterization. The schematic illustrates structural differences, common direct and indirect characterization methods, and the influence of mechanical properties on applications such as drug release, imaging, and acoustofluidic targeting.

The characterization methods of the mechanical properties of microbubbles (MBs) and echogenic liposomes (ELIPs) can be categorized into two groups (Figure 1). Direct characterization methods measure the deformation of MBs and ELIPs due to mechanical loading. These include optical observation of the agent’s radius dynamic response to sonication and atomic force microscopy (AFM), which measures deformation under applied compression force. Optical observation of MB/ELIP diameter changes under sonication can reveal how shell elasticity responds to acoustic pressure. Although these methods provide high-resolution insights into individual MBs or ELIPs, they can be time-consuming and may not represent population-level variability.

Indirect methods, in contrast, estimate mechanical properties by analyzing how MBs or ELIPs affect external signals, typically acoustic or optical. Frequency-dependent attenuation techniques measure the attenuation of an ultrasonic wave passing through an MB/ELIP suspension, and from the attenuation spectra, shell stiffness and friction parameters can be extracted based on theoretical models (e.g., modified Rayleigh–Plesset). Laser scattering is another indirect approach, detecting changes in scattered light intensity that correlate with radial oscillations of MBs or ELIPs. While these methods generally accommodate larger sample volumes and expedite data collection, assumptions about linear oscillation or monodisperse size distributions can introduce uncertainties.

There have been several reviews on the use of MBs and liposomes as drug delivery vehicles focusing on their formulations and chemical compositions [4,5], biosafe use practice [6,7], shell radial dynamic response [8,9,10], and potential applications [10,11,12,13,14,15,16,17,18,19,20,21]. However, this review focuses solely on mechanical properties of these nano-carriers and the methods that characterize them. A conceptual overview of the structural differences between microbubbles and echogenic liposomes, the characterization methods used to evaluate them, and their medical applications is illustrated in Figure 1.

This review presents an overview of the mechanical characterization of medical microbubbles and echogenic liposomes (ELIPs), with a particular focus on their modeling, experimental measurement, and temperature-dependent behavior. The models commonly used to predict mechanical properties are reviewed first, followed by the characterization techniques implemented to estimate shell stiffness and shell viscosity. A comparative discussion of the factors that influence the measurement outcomes is provided, along with a review of temperature and composition effects. The performance of ELIPs is evaluated in comparison to commercial microbubble formulations. The objective is to provide a structured overview of the current understanding of shell mechanics and their implication in developing acoustically responsive delivery agents.

## 2. Historical Overview of the Major Developments in Modeling of Microbubble Physics

**Figure 2:**
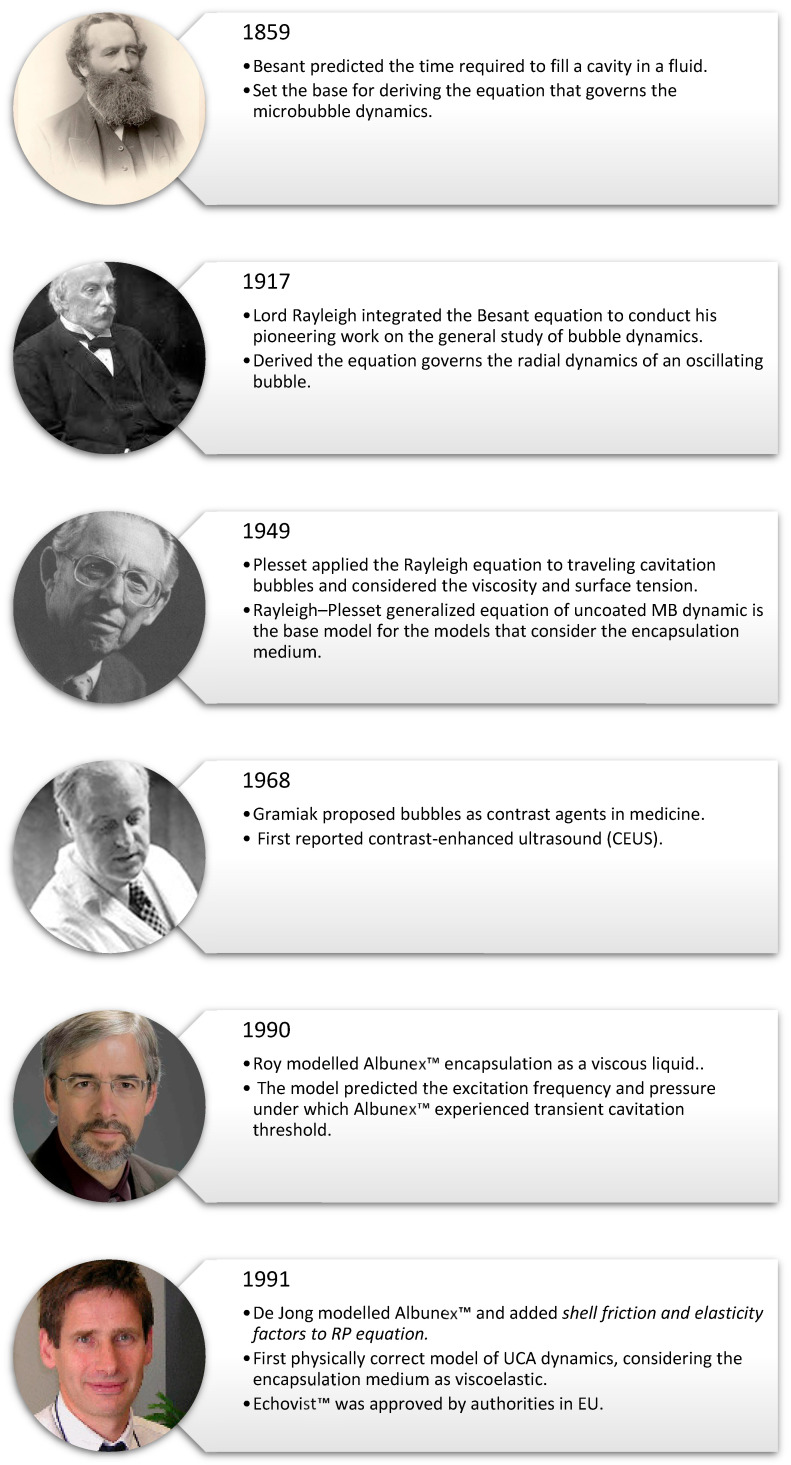
History of microbubble science, presented in chronological order of research developments.

An overview of the history of MB science is presented in Figure 2. In 1859, William Henry Besant was the first scientist to document the time required to fill a cavity in an incompressible fluid, neglecting viscosity, surface tension, and pressure variant inside the bubble [22]. Besant derived the root of the equation that governs the bubble dynamic—an equation later known as the Rayleigh–Plesset equation. Interest in the dynamics of MBs grew when the British Royal Navy started investigating rapid propeller erosion in its warships in the late nineteenth century. Propeller damage caused by bubble cavitation prompted studies in 1917 by John William Strutt, better known as Lord Rayleigh, into the implosion of spherical cavities in liquids [23]. Rayleigh realized that pressure inside the cavity is not constant. Using Boyle’s law, Raleigh showed that when the cavity decreases by a specific factor, the pressure near the boundary of the cavity becomes greater than the ambient pressure [24]. Rayleigh considered the bubble at the center of a spherical coordinate system where the only possible motion is radial, i.e., the bubble is oscillating but not translating. Neglecting the surrounding fluid viscosity, surface tension, and thermal effects, Rayleigh derived the equation for the oscillation dynamics of a gas-filled cavity in an incompressible fluid. In 1949, Milton S. Plesset included the viscosity and the surface tension in the equation and applied it to a traveling cavity [25]. One form of the Rayleigh–Plesset (RP) equation and its assumptions are shown in the next section, Section 3.1. The equations that govern the dynamics of MBs and consider different MB encapsulation mediums are modified models based on Rayleigh–Plesset equation [26,27,28,29,30], and some of the modification and assumptions are mentioned briefly in Section 3.1. These models are used to estimate the shell elastic properties of MBs and ELIPs.

Decades later, in 1968, Gramiak and Shah first reported the use of small bubbles in enhanced contrast ultrasound imaging [31]. The UCAs were used to enhance the reflected ultrasonic signal from the aortic valve. The first model that included the encapsulation of UCA and considered a viscous liquid shelling medium was developed by Roy in 1990 [32]. Roy’s model predicted the acoustic excitation parameters under which Albunex™ exceeded the transient cavitation threshold, and the model compared favorably with the experimental results. A viscoelastic shell assumption was then considered by De Jong, which included shell elastic and friction factors to the RP equation to characterize Albunex™, the first clinically approved UCA [33]. De Jong’s model was the first model describing the encapsulation medium of UCA as viscoelastic. These aforementioned pioneering studies set the base for the investigations on the mechanical behavior of ultrasonic contrast agents and acoustofluidic delivery agents, which are used in the thesis.

## 3. Radial Dynamic Response

The models that predict the MBs oscillations are essential to understanding their acoustic behavior and optimizing formulations for imaging or drug delivery. This section outlines the theoretical models commonly used to describe shell dynamics. An MB exposed to an acoustic wave oscillates in different modes depending on its mechanical properties and the characteristics of the excitation wave. The behavior of a bubble in an infinite three-dimensional space is relatively well understood and described in detail in Leighton’s book *The Acoustic Bubble* [34], and in a simplified elaboration in Richard Manasseh’s work [35]. The radial dynamic of the bubble is governed by the Rayleigh–Plesset equation, which underwent several modifications to include shelled bubble parameters. A detailed and comprehensive history of the development of the models and their assumptions of the shell is found in [8,9,10], and some of them will be discussed briefly in the next section. Measurements of radial dynamic response to specific mechanical loading allows a calculation of the mechanical properties of the MBs’ and ELIPs’ shells. These properties directly affect the agent’s acoustofluidic behavior.

### 3.1. Rayleigh–Plesset Equation

One form of the Rayleigh–Plesset equation governing an uncoated MB is given as in [24,25], as follows:

(1) $$\rho_{L}\left(R\ddot{R}+\frac{{\dot{3R}}^{2}}{2}\right)=P_{v}-\frac{2\sigma }{R}-\frac{4\eta \dot{R}}{R}-P_{0}+P_{a}\left(t\right)$$

where $\rho_{L}$ is the density of the liquid, R is the instantaneous radius, $\dot{R}$ is the first-order time derivative (radial velocity),$\;\ddot{R}$ is the second-order time derivative (radial acceleration), $P_{v}$ is the gas pressure inside the bubble, σ is surface tension, $\eta_{L}$ is viscosity of the fluid, $P_{0}$ is the ambient pressure, and $P_{a}\left(t\right)$ is the time-dependent excitation pressure. This nonlinear equation describes the time-dependent radius of a sonicated bubble. Although it was thought that there was no analytical solution to the Rayleigh equation [36], a closed-form general solution of the Rayleigh equation for empty and gas-filled bubbles was devised in 2014 by Kudryashov and Sinlshchikov [37]. The solution agreed well with experimental data and numerical solutions at specific polytrophic exponents.

The assumptions for the RP equation are as follows:

- The MB is filled with a compressed ideal gas.
- The MB motion is symmetrical.
- The wavelength of the excitation wave is much longer than the MB radius.

There is no rectified diffusion between the MB and the surrounding fluid. Rectified diffusion is a phenomenon that describes the exchange of gas between sonicated bubbles and the surrounding fluid. The pressure inside an oscillating bubble changes—decreases as it expands and increases as it compress—which may results in consequent gas diffusion in and out of the bubble, which is called rectified diffusion [38].

Equation (1) can be linearized and expressed in the linear harmonic oscillator form to obtain the damping coefficient and the resonance frequency, as demonstrated in [10]. The resonance frequency can be used to estimate the shell stiffness and the shell damping coefficient is used to estimate the friction coefficient of the MB. The time-dependent radius solution of the linearized form can be used to calculate the primary radiation force, as shown in [2], which results in MBs’ and ELIPs’ global movement due to sonication.

#### Modified Rayleigh–Plesset Equation

The Rayleigh–Plesset (RP) equation has undergone several modifications to incorporate the effect of the shell on the MB dynamics [26,27,28,29,30], and a detailed and comprehensive history of the development of the models and their assumptions of the shell are found in [8,9,10]. Some models assume incompressibility in the liquid or at the interface; however, these assumptions are intuitively incorrect as sound waves require compressibility to exist. Although, incompressibility could provide a valid assumption for simplifying calculations as the compressibility of the shell and liquid is negligible. This contrasts with the high compressibility of the filling gas. A model that considers compressibility and viscoelasticity of the surrounding liquid confirmed the minimal effect of the shell and liquid on the radial dynamic response of bubbles [30].

The first rigorous theoretical modification to include the encapsulation medium in the RP equation was performed by Church [29]. The model considered three regions—gas, shell, and liquid—where a layer of incompressible viscoelastic shell with damping separating the bulk Newtonian liquid and the gas is considered. In this section, some of the modified RP equations are described, as they are used to characterize MBs and ELIPs in the following sections.

Hoff model

Building on Church’s model and assuming that the shell thickness is much smaller than the shell radius, Hoff simplified the model and derived Equation (2) [3]:

(2) $$\rho_{L}(R\ddot{R}+\frac{3}{2}{\dot{R}}^{2})=P_{0}{\left(\frac{R_{0}}{R}\right)}^{3\gamma }-4\eta_{L}\frac{\dot{R}}{R}-12\eta_{s}\frac{d_{s}R_{0}^{2}}{R^{3}}\frac{\dot{R}}{R}-12G_{s}\frac{d_{s}R_{0}^{2}}{R^{3}}\frac{\dot{R}}{R}\left(1-\frac{R_{0}}{R}\right)-P_{0}+P_{a}\left(t\right)$$

where γ is the polytropic exponent of the gas, $R_{0}$ is the ambient bubble radius,$\;\eta_{s}$, is the shell viscosity, $d_{s}$ is the shell thickness, $G_{s}$ is the shell shear modulus, and the other symbols are the same as mentioned previously.

Sarkar model

In 2003, Chatterjee and Sarkar argued that the encapsulation media typically consist of a few layers of molecules and cannot be considered to be a homogenous media with bulk material properties [39]. Instead, Chatterjee and Sarkar proposed an interfacial Newtonian rheological model that considered the encapsulation media as a zero-thickness surface with complex interfacial properties. The model can be used for calculating the dilatational viscosity and interfacial tension:

(3) $$\rho_{L}\left(R\ddot{R}+\frac{{\dot{3R}}^{2}}{2}\right)=\left(p_{0}+2\frac{\sigma_{i}}{R_{0}}\right){\left(\frac{R_{0}}{R}\right)}^{3\gamma }-4\eta_{L}\frac{\dot{R}}{R}-2\frac{\sigma_{i}}{R}-4\frac{\kappa_{s}\dot{R}}{R^{2}}-p_{0}-P_{a}\left(t\right)$$

where $\sigma_{i}$ is the interfacial tension and $\kappa_{s}$ is the dilation viscosity.

Marmottant model

Marmottant considered the shell of a monolayer lipid MB as a two-dimensional viscoelastic medium. The model assumes that the surface tension of phospholipid MB shell is divided into three regimes: buckling state, for which buckling reduces surface tension to zero; elastic state, where the surface tension is linearly dependent on the MB radius; and rupture state, above a specific radius where the surface tension is the same as at a standard water–air interface. Marmottant’s modified RP equation is as follows:

(4) $$\rho_{L}\left(R\ddot{R}+\frac{3}{2}{\dot{R}}^{2}\right)=\left(p_{0}+2\frac{\sigma_{w}}{R_{0}}\right){\left(\frac{R_{0}}{R}\right)}^{3\gamma }\left(1-\frac{3\gamma }{c}\dot{R}\right)-4\chi \left(\frac{1}{R_{0}}-\frac{1}{R}\right)-2\frac{\sigma_{w}}{R}-4\eta_{L}\frac{\dot{R}}{R}-4\frac{\kappa_{s}\dot{R}}{R^{2}}-p_{0}-P_{a}\left(t\right)$$

where χ is the compression modulus of elasticity, $\sigma_{w}$ is the surface tension of the liquid, and $\kappa_{s}$ is the dilation viscosity.

While these models provide a theoretical basis for understanding shell behavior, their assumptions must be evaluated against experimental data. The next section reviews experimental techniques used to characterize mechanical properties under physiologically relevant conditions.

## 4. Characterization Methods

Microbubbles’ shell properties have a significant effect on the microbubble’s volumetric oscillation and translational velocity. These properties can be measured directly through mechanical loading and measurement of deformation, or indirectly through ultra-sonicating the MBs’ and ELIPs’ suspension and measuring the radial-dynamic response optically or measuring the change in the acoustic wave behavior through attenuation or scattering measurement. The following subsections provide an overview of the most frequently used characterization methods and their relevance to both experimental validation and clinical application.

### 4.1. Frequency-Dependent Attenuation

**Figure 3:**
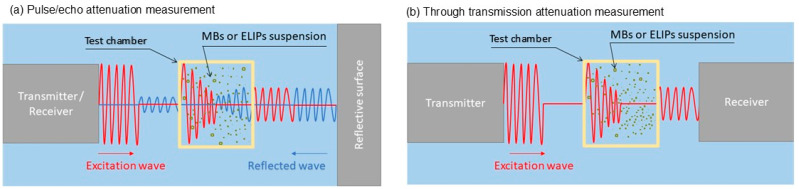
Schematic of frequency-dependent attenuation measurement techniques for microbubble (MB) and echogenic liposome (ELIP) suspensions. (**a**) Pulse/echo method: the excitation wave reflects off a surface, with attenuation measured from the returning signal. (**b**) Through transmission method: attenuation is measured from the transmitted wave detected by a separate receiver.

One of the methods to characterize the shell parameters is through measuring the frequency-dependent attenuation of acoustic beam through a suspension of MBs or ELIPs. This could be achieved by sending a pressure wave through the agents’ suspension and measuring the transmission loss after the wave passes through the suspension. This could be performed using (a) a pulse echo technique, where the pressure wave is reflected to be acquired by the same excitation transducer (Figure 3a), and (b) through transmission, where another transducer or preferably hydrophone is used to acquire the attenuated wave after it has passed the suspension (Figure 3b).

The measurement of frequency-dependent attenuation factor can be used to estimate the shell elastic properties as described in details in [40]. Attenuation measurements can be obtained by measuring the transmission loss of acoustic wave through a suspension of MBs and ELIPs. The experimental attenuation factor can be calculated through relative measurements of the wave pressure without the presence of the sample $P_{i}$ and with the presence of the sample $P_{b}\;$, in an attenuation path of the length L using Equation (5):

(5) αexp=20log10PiPbL

The attenuation factor $\alpha \left(r,f\right)$ of microbubbles of radius r at frequency f is found as

(6) $$\alpha \left(r,f\right)=\frac{10}{ln(10)}*\sum_{r}n\left(r\right)*\sigma_{e}\left(r,f\right)$$

where $n\left(r\right)$ is the number density of the microbubbles that can be obtained by any particle counting technique and $\sigma_{e}\left(r,f\right)$ is the extinction cross-section denoting the total energy loss from the acoustic beam traveling through the bubble, found as

$$\sigma_{e}=\sigma_{s}+\sigma_{a}$$

where $\sigma_{s}\left(r,f\right)$ is the scattering cross-section shown by Medwin [41]:

(7) $$\sigma_{s}\left(r,f\right)=\frac{4\pi r^{2}}{{\left\{{\left(\frac{f_{0}\left(r\right)}{f}\right)}^{2}-1\right\}}^{2}+\delta_{tot}^{2}(r,f)}$$

Here, $\sigma_{a}\left(r,f\right)$ is the absorption cross-section describing the acoustic energy loss as heat convection, which can be found in [42] as

(8) $$\sigma_{a}\left(r,f\right)=\sigma_{s}\left(\frac{\delta_{tot}}{\delta_{rad}}-1\right)$$

The total damping $\delta_{tot}$ can be approximated as the summation of damping due to radiation $\delta_{rad}$, viscosity $\delta_{vis}$ and shell friction $\delta_{sh}$, such as

(9) $$\delta_{rad}=\frac{\omega R}{c}$$

(10) $$\delta_{vis}=\frac{4\eta }{\omega \rho R^{2}}$$

(11) $$\delta_{sh}=\frac{S_{f}}{4\pi \omega \rho R^{3}}$$

where ω  is the angular frequency (2πr), η is the viscosity of the liquid, and ${\;S}_{f}\;$ is the shell friction coefficient. The resonance frequency derived considering small radial oscillation with respect to the resting radius and assuming the shell is a viscoelastic solid was found in [43] as

(12) $$f_{0}=\frac{1}{2\pi }\sqrt{\frac{3\gamma P_{0}}{\rho R^{2}}+\frac{2S_{P}}{\rho R^{3}}}$$

where γ  is the polytropic exponent of the contained gas, $P_{0}$ is the ambient pressure, ${\;S}_{p}$ is the shell stiffness coefficient, and ρ is the liquid density. Other models with different assumptions regarding the shell medium derive similar resonance frequency equations [44].

The models developed to estimate the elastic properties of microbubble shell and the previous models they are built on were derived applying critical assumptions: namely, (1) small radial oscillation is relative to the resting radius [43]; (2) bubbles are oscillating linearly; (3) bubbles are behaving as individual entities [41], not as clusters; and (4) the bubbles’ radial responses are due to the primary acoustic source, not due to acoustic influence from other bubbles due to secondary Bjerkness force. Consequently, this requires the bubbles to be relatively far from each other, i.e., suspended in a high dilution.

The assumptions are hard to achieve experimentally. Studies performed on individual BR-14 (Bracco S.A., Geneva, Switzerland) microbubbles, sonicated at 4–13.5 MHz, showed that they undergo nonlinear behaviors at acoustic pressures as low as 13 kPa [45] and 12.5 kPa [46]. Using frequency-dependent attenuation factor measured from MBs oscillating nonlinearly could result in an invalid calculation of shell elastic properties, as the model is not designed to account for nonlinear oscillation. The pressure to excitation voltage variance is also an inevitable transducer limitation. This highlights the limitation of linearized Rayleigh–Plesset-based models, which underestimate shell compliance during large amplitude oscillations. In such scenarios, a model like Marmottant’s—which accounts for buckling and rupture of lipid shells—would be more suitable for capturing the dynamic mechanical response. A recent study used constant pressure by opposing the transducer pressure with the excitation voltage curve [47]; through measuring the voltage, a desired acoustic pressure in the test chamber is produced.

Pressure-dependent attenuation is another crucial factor that should be avoided when investigating frequency-dependent attenuation, as microbubbles have been shown to have a significant pressure-dependent attenuation factor [48,49,50], and the model does not account for pressure. The pressure-dependent attenuation influence on the shell property estimation can be observed in Table 1, where higher excitation pressure increases the shell stiffness estimation over approximately similar frequency ranges. Pressure magnitude could affect the mode of vibration of the microbubbles and cause the translation of microbubbles and, therefore, cluster formation. Studies published earlier found that individual microbubbles sonicated at 4–13.5 MHz can undergo nonlinear behaviors at acoustic pressures as low as 13 kPa [45]. A threshold pressure of microbubble translation was observed for single MB [51] and MB populations [52]. Definity^®^ MBs were shown to have a significant translation and clusters formation at pressures as low as 6 kPa.

In 2003, Chatterjee and Sarkar argued that the encapsulation medium typically consists of a few layers of molecules and hence cannot be considered a homogenous medium with bulk materials properties [39]. So, they proposed an interfacial Newtonian rheological model considering the encapsulation medium as a macroscopic homogeneous continuum. The model can be used for calculating the dilatational viscosity and interfacial tension. They used the attenuation curves and size distributions of Albunex™, Optison™, and Quantison™ [33,56,57] and produced them for Sonazoid™ in [58] to calculate the dilation viscosity $\kappa^{s}$ and interfacial tension γ of the agents, as summarized in Table 2.

The properties estimated using these methods need to be interpreted with caution, as the models attempt to derive the properties of a single microbubble from a population of microbubbles. The frequency–radius-dependent response of a population of microbubbles with various sizes will always be a challenge to ascertain, no matter how the accuracy of the measurement is. Best practice is to use a monodisperse sample in the attenuation measurements, as in [55]. However, this ideal dispersity achieved in the lab is not practical for clinical use, as it is hard to maintain.

For ELIPs suspensions, a considerable number of them are not echogenic, which means they do not contribute to the attenuation measurement. That is because phospholipids have an acoustic impedance approximately equal to the impedance of human tissue and water [59], and therefore do not reflect ultrasonic waves. To account for that, Equation (3) can be modified, as in [55], to be

(13) $$\alpha \left(r,f\right)=\frac{10}{ln(10)}*N_{fit}\sum_{r}n_{k}(r)*\sigma_{s}\left(r,f\right)*\frac{\delta_{tot}}{\delta_{rad}}$$

where $N_{fit}$ is the total number of bubbles per unit volume and $n_{k}$ is the normalized number distribution. In this form, the value of $N_{fit}$ is calculated from the attenuation measurement, not from the size distribution measurement.

In the latter equation, the $N_{fit}$ could be used as an estimation for the ELIPs and the percentage of the non-echogenic liposomes could be calculated by subtracting $N_{fit}$ from the total number of the liposomes measured using one of the concentration and size distribution measurement techniques like coulter counter and Tunble Resistive Pulse Sensing. For more accurate assessment, resonance mass measurement (RMM) could also be used to distinguish ELIPs and MBs from non-echogenic liposomes [60]. Where non-echogenic liposomes are expected to have higher density, as ELIPs of a similar size having a fraction of their volume filled with gas will have a lower density.

Compared to AFM and optical methods, acoustic attenuation measurements allow the non-invasive estimation of average mechanical properties over a large microbubble population under realistic insonation conditions. However, they are less suitable for probing shell heterogeneity or capturing local variations, and rely heavily on fitting assumptions within theoretical models, such as the choice of shell viscoelastic parameters and pressure conditions.

### 4.2. Optical Acquisition

**Figure 4:**
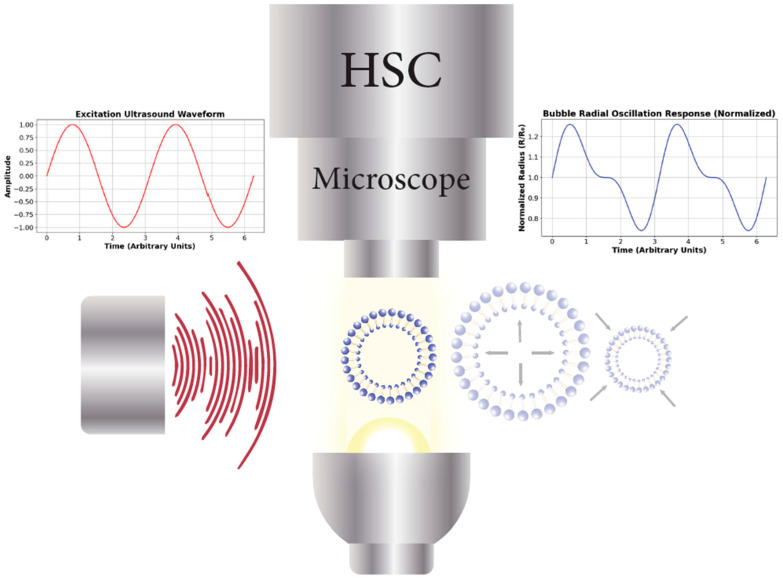
Schematic representation of the optical acquisition setup for studying ultrasonicated microbubbles (MBs) and echogenic liposomes (ELIPs). A high-speed camera (HSC) microscope captures the dynamic radial oscillations of individual bubbles in response to.

Direct optical observation has been used to study ultrasonicated MBs [61] and ELIPs [62,63], enabling the real-time tracking of their volumetric dynamic response, radial oscillations, deformation, and rupture behavior. High-speed imaging tracks MBs’ [64,65] or ELIPs’ [63] shape changes under applied acoustic or mechanical forces, quantitatively estimating shell stiffness and viscoelastic properties based on bubble deformation relative to the known stresses (Figure 4). Studies evaluating MB and ELIP shell properties via optical observation are summarized in Table 3.

Morgan et al. characterized the shell of the experimental agent MP1950 (Mallinckrodt^®^, Saint Louis, MO, USA) [61]. They modified the Herring equation, which is another model that describes MBs’ radial dynamics due to sonication. They argued that the model considering the losses in separate terms has an advantage over the RP equation in predicting MB’s radius fluctuations over 100%. Their results showed independency between size and shell elasticity (Table 3), and that the viscosity is linearly correlated to the MB radius. Their study also highlighted the critical role of bubble size, showing that smaller microbubbles (<1.0 µm radius) exhibit higher-frequency shifts and faster ring-down, while larger bubbles (>1.8 µm radius) produce stronger echoes with slower ring-down, underscoring the importance of size distribution in optimizing contrast agent performance.

The last three rows of the table show an increasing shell compression elastic modulus with radius, which does not agree with atomic force microscopy results (Section 4.4) and frequency-dependent attenuation results (Section 4.1). This might suggest that the ELIPs have different compression modulus than the expansion elastic modulus, as the elasticity described in the Marmottant model is compression elasticity. This phenomena has been observed using atomic force microscopy, where the ELIPs made of DSPC:DSPE-PEG2000:DSPE-PEG2000-CH at a molar ratio of 90:5:5 showed higher deformation to the applied load during loading and lower deformation to the applied load during unloading [66].

The reduction in stiffness with increased temperature of Sonovue™ MBs was deduced from optical observation of their oscillation. Sonovue™ MBs showed up to a two-fold increase in radial amplitude at body temperature when excited with acoustic pressure 40 kPa, and Definity™ MBs of 3 µm diameters showed increased radial oscillation in comparison to room temperature [67]. The study is not included in the table because it only reports the radial expansion due to sonication, without calculating the elastic properties. However, increased radial oscillation at increased temperature when all the other parameters are held constant indicates a reduction in the elasticity.

**Table 3:** Summary of previous studies reporting shell properties of microbubbles and echogenic liposomes measured through optical observation.

Study	Agents	P (kPa)	f (MHz)	Model	Radius (µm)	Elasticity	Viscosity
[61]	MP1950	310	2.4	Morgan et al.	2.6	χ = 0 N/m	$\epsilon \eta_{s}$ = 4 nm Pa s
		360	2.4		2.6	χ = 1.1 N/m	$\epsilon \eta_{s}$ = 3.4 nm Pa s
		360	2.4		1	χ = 0 N/m	$\epsilon \eta_{s}$ = 0.6 nm Pa s
[68]	BR-14	≤40	2.5	Marmottant	1.5–5.2	χ = 0.5 N/m	$k_{s}(R_{0})\;=\;2.5\times 10^{-9}$ $5.5\times 10^{-9}$ kg/s *
[68] fitted by [69]	BR-14	40	2.5	Marmottant	1.7	χ = 0.25 N/m	$k_{s}\;=\;4\times 10^{-9}\;kg/s$
				Sarkar		$\sigma_{i}\;=\;0.32\;N/m$	$k_{s}\;=\;4\times 10^{-9}\;kg/s$
				Hoff		$G_{s}\;=\;23\;MPa$	$\eta_{s}\;=\;0.5\;Pa\;s$
[63]	EggPC:DPPC:DPPE:DPPG:CH (27:42:8:8:15)-(Air)	250		Marmottant	1.5	χ = 0.1 N/m	$k_{s}\;=\;3.3\times 10^{-9}\;kg/s$
			4		2.3	χ = 0.2 N/m	$k_{s}\;=\;6.0\times 10^{-9}\;kg/s$
					3	χ = 1.55 N/m	$k_{s}\;=\;2.15\times 10^{-9}\;kg/s$
[67]	Sonovue™	40–80	0.5	Hoff	6.5	χ = 0.048 N/m	
					8.1	χ = 0.228 N/m	
					9.3	χ = 0.36 N/m	
					10.3	χ = 0.51 N/m	
					11	χ = 0.66 N/m	

P is the excitation pressure, χ is the shell compression modulus of elasticity, $k_{s}$ is the dilation viscosity, $\sigma_{i}$ is the interfacial tension, $G_{s}$ is the shear modulus, $\eta_{s}$ is the shear viscosity, and ϵ is the shell thickness. * a radius-dependent value.

Visually capturing bubble expansion, contraction, coalescence, and breakup requires shutter speeds ranging from tens to hundreds of nanoseconds. Capturing bubble cavitation requires shutter speeds of mere picoseconds [16]. The main problem when using high-speed cameras is the inverse relationship between spatial resolution and frame rate. That is, if frame rate increases, spatial resolution decreases [70]. Another disadvantage of using high-speed photography is the required lighting setup that might introduce undesirable and variable factors, such as heat. High frame rates also produce large numbers of frames, ranging from several hundreds to millions of frames per seconds, that require significantly large data storage capabilities and high-performance computers to process the data.

Compared to AFM or acoustic attenuation, optical acquisition techniques provide dynamic, real-time data at the individual bubble level. However, they are limited by optical access, field of view, and are not easily applicable to in vivo or turbid media, restricting their use to well-controlled in vitro settings.

### 4.3. Light Scattering

In 2004, Guan [71] attempted to evaluate the feasibility of measuring the individual pulsating bubble using light scattering. He argued that the data acquired using a high-speed camera is limited to a few acoustic cycles due to the storage space requirements discussed in the latter section. The experiment subjected Optison™ and Sonazoid™ to pulsed ultrasound. Using 30 mW HeNe and a 100 µm focused-beam laser, the microbubbles were illuminated, and the scattered light was then focused into a photomultiplier tube PMT. The received signal is then related to the bubble radius and the bubble response to sonication was studied in a gummy medium (Xanthan gum powder, glycol, and water).

There are several points worth noting in this experiment. The process to isolate a single bubble assumes that a highly diluted UCA solution with a defined discharge rate will result in a uniform flow of bubbles. However, there is no logical reason that guarantees that the bubbles will discharge uniformly one by one. The liquid containing the bubbles might discharge without bubbles. This is why the calculated bubble flow rate that was supposed to detect a scattering event every 3 ms reportedly failed. Another disadvantage of this technique is that the medium containing the bubbles has high viscosity, which will affect the shell–liquid interface properties.

Table 4 summarizes several studies used to characterize several MBs and ELIPs that showed acceptable agreement with optical and acoustical results published prior.

In contrast to AFM and acoustic attenuation, light scattering techniques provide high temporal resolution and are well suited to studying oscillatory dynamics in freely suspended microbubbles. However, they often require assumptions about optical symmetry and refractive index matching and typically yield indirect estimates of mechanical properties that must be inferred from model fitting rather than measured directly. The reliability of such inferences depends not only on the chosen model but also on how accurately the input parameters reflect the experimental environment. For example, neglecting temperature effects or medium viscosity in fitting may shift the estimated stiffness significantly.

### 4.4. Atomic Force Microscopy

**Figure 5:**
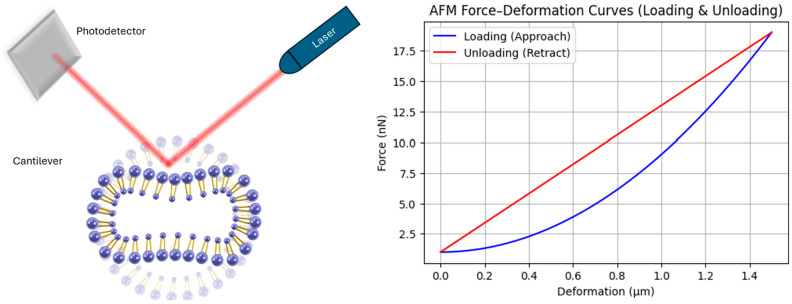
Experimental AFM setup for microbubble compression (**left**) and the corresponding loading–unloading force–deformation curves (**right**), showing how the ratio of deformation to force can differ between approach and retract.

The elastic properties of the agents could be measured directly by applying a compression force on a single agent using atomic force microscopy (AFM) (Figure 5, left), and correlating the deformation to the applied load during both the loading (approach) and unloading (retract) phases, generating characteristic force-deformation curves (Figure 5, right). The difference between loading and unloading curves is indicative of viscoelastic behavior and hysteresis effects within the bubble shell, highlighting the complex mechanical response of lipid-coated microbubbles. Unlike the scanning electron microscopy, the atomic force microscope does not require subjecting the sample to low pressure and temperature. This is an important advantage in the case of investigating microbubbles, as their pressure–volume change is significant.

The atomic force microscope has been used to mechanically load lipid-shelled microbubbles to investigate their stiffness. Studies have found that the mean value of the stiffness of Biosphere™ MBs of different sizes is between 1 and 6 N/m, with smaller MBs being stiffer than larger ones [73]. Another investigation found that the presence of charged lipids reduces ELIP stiffness by 30% to 60%, when compared to those formulated with neutrally charged lipids [74].

**Figure 6:**
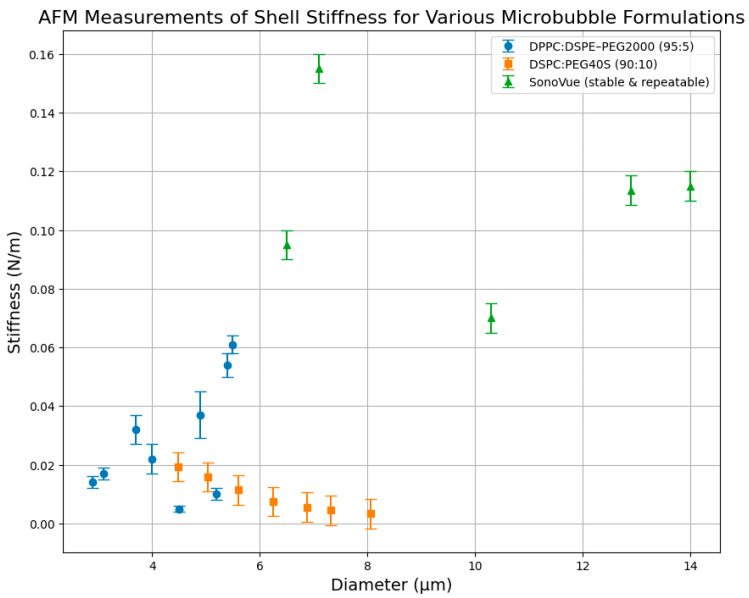
AFM measurements of shell stiffness for different lipid-shelled microbubble formulations. The stiffness (N/m) was measured as a function of bubble diameter (µm) for DPPC:DSPE-PEG2000 (95:5) microbubbles [75], DSPC:PEG40S (90:10) microbubbles [66], and SonoVue™ microbubbles (stable and repeatable measurements) [76].

Several studies have utilized AFM to measure the size-dependent stiffness of lipid-shelled microbubbles (Figure 6). Shafi et al. [75] performed measurements on DPPC:DSPE-PEG2000 (95:5) microbubbles, reporting stiffness values between 0.014 and 0.061 N/m for diameters ranging from approximately 2.9 to 5.5 µm. Their results illustrate notable variability and a general trend of increasing stiffness with bubble size, suggesting that complexities in shell mechanics are potentially influenced by microstructural heterogeneity or measurement uncertainties.

Chen et al. (2013) [66] presented stiffness measurements for DSPC:PEG40S (90:10) microbubbles, clearly illustrating a significant size-dependent behavior. Their stiffness data (Figure 6) showed an exponential decay from around 0.0193 N/m for smaller microbubbles (~4.5 µm), down to 0.0045 N/m for larger microbubbles (~7.3 µm), emphasizing an inverse correlation between stiffness and diameter. These findings align with theoretical expectations, supporting the notion that larger microbubbles have softer shells due to decreased shell curvature stresses.

In contrast, Chetty et al. (2008) [64] investigated SonoVue microbubbles and identified stable and repeatable stiffness measurements within a wider size range (~6.5–14 µm). Their results exhibited stiffness values between 0.070 and 0.155 N/m. Interestingly, SonoVue bubbles did not exhibit a clear monotonic trend but rather displayed size-dependent stiffness variability. This might be attributed to structural heterogeneities in the SonoVue formulation or experimental variations.

Compared to acoustic attenuation or optical methods, AFM offers localized mechanical probing with nanonewton precision, but is limited by its low throughput, the need for surface immobilization, and its difficulty in capturing real-time dynamic responses under acoustic excitation.

While each method offers specific advantages, no single technique fully captures the spatial, temporal, and environmental complexity of microbubble and liposome mechanics. Future progress may be driven by multimodal characterization approaches that combine mechanical testing with optical, acoustic, or molecular imaging techniques. For instance, integrating AFM with high-speed imaging or combining acoustic backscatter measurements with fluorescence microscopy can yield complementary information on shell deformation and gas core behavior. In parallel, high-throughput platforms using microfluidics or automated indentation arrays could enable population-level mechanical profiling with improved statistical power. The use of AI or machine learning to infer viscoelastic properties from indirect measurements may also provide a pathway toward rapid, standardized characterization protocols.

## 5. Effect of Temperature on Shell Elastic Properties

As shown in Table 1, the stiffness of monolayer MBs decreases as temperature increases. However, frequency-dependent attenuation measurements are not conclusive in deciding the change in stiffness with temperature, as the size distribution also changes with temperature [47] and size distribution directly affects the stiffness estimations. The reduction in stiffness with increased temperature is also confirmed with optical observation of their oscillation. Sonovue™ MBs showed an up to two-fold increase in radial amplitude at body temperature when excited with acoustic pressure 40 kPa and Definity™ MBs of 3 micron diameters showed increased radial oscillation in comparison to room temperature [67]. The threshold pressure of MBs oscillation is higher at room temperature [67], which could also be an indication of a stiffer shell at room temperature in comparison to physiological temperature.

Frequency-dependent attenuation measurements unexpectedly showed that ELIPs have an increase in their stiffness as the temperature increases to physiological temperature, as in Table 1. The stiffness increased from 1.13 N/m at 25 °C to 1.49 N/m at 37 °C for ELIPs made of L-α-phosphatidylcholine (EggPC), 1,2-dipalmitoyl-*sn*-glycero-3-phosphoethanolamine (DPPE), 1,2-dipalmitoyl-*sn*-glycero-3-phospho-[1′-rac-glycerol] (DPPG), and cholesterol (CH), at a molar ratio of 69:8:8:15; from 1.98 N/m at 25 °C to 3.10 N/m at 37 °C for ELIPs made of EggPC:DPPC:DPPE:DPPG:CH, at a molar ratio of 27:42:8:8:15; and from 2.05 N/m at 25 °C to 4.06 N/m at 37 °C for ELIPs made of DPPC:DOPC:DPPG:CH, at a molar ratio of 46:24:24:6 [55]. Similarly, it increased from 0.11 N/m at 25 °C to 0.15 N/m at 37 °C for ELIPs made of DPPC:DSPE-PEG2000, at a molar ration of 94:6. However, as discussed in the section regarding frequency-dependent attenuation, these measurements have several limitations, specially for ELIPs if the ratio of the echogenic to non-echogenic liposomes is unknown or estimated from attenuation curves, as in the presented studies.

Overall, increasing temperature generally leads to reduced shell stiffness and increased deformability, consistent with lipid phase transitions and reduced shell viscosity observed in both AFM and attenuation-based measurements. However, discrepancies persist across studies, particularly in the magnitude of temperature sensitivity and the role of shell composition. For example, some formulations exhibit minimal mechanical change between 25 °C and 37 °C, while others show pronounced softening. These differences may arise from variation in lipid packing, gas solubility, measurement method, or acoustic parameters. Additionally, ELIP structures introduce complexity due to their bilayer organization and heterogeneous gas encapsulation, making direct comparison with MBs challenging. As a result, while the general direction of thermal and compositional influence is clear, further studies are needed to unify these effects within a comprehensive model of shell mechanics that accounts for both thermodynamic state and experimental modality.

## 6. Effect of Chemical Composition on Shell Properties

Liposome shells could be stiffened by adding cholesterol [75] and/or poly-ethylene glycol (PEG)-grafted phospholipids [76] to their composition. Lipid-coated MBs with longer hydrophobic chains increase MBs’ permeation of hydrophobic gasses which consequently enhances their stability [77]. PEG coating also enhances their stability against aggregation and coalescence [78], if the zeta potential of the MBs is not significant for their aggregation stability. For example, Definity™ has a zeta potential of −4.2 mV and yet it is stable due to the PEG graft. Another study measured the zeta potential of Definity™ to be −2.43 ± 0.54 mV, using tunable resistance pulse sensing, and −0.62 ± 0.26 mV using phase analysis light scattering [79]. The low zeta potentials of PEGylated agents, as confirmed by TRPS and PALS measurements, highlight the dominance of steric stabilization over electrostatic repulsion in preventing aggregation.

The zeta potential of ELIPs could be controlled by varying their formulating phospholipids [80,81], where increasing the negatively charged phospholipid in the composition results in more negative zeta potential and vice versa. Similarly, MBs consisting of more negatively charged lipids have shown more negative zeta potential [82].

The effect of zeta potential and the charges of the phospholipids composing the MBs and ELIPs on the shell stiffness is not yet clear. Neutral phospholipids have been shown to increase the stiffness of ELIPs, as their molar ratio increases relative to the charged phospholipids in [74], while the presence of negatively charged DOPS was also shown to increase the ELIPs stiffness [76,83]. Future studies on these aspects performed with same experimental setups for different MBs and ELIPs of different compositions could reveal a correlation between shell elastic properties and their composing lipids. The mechanical properties of microbubbles are largely determined by their lipid shell composition. The selection of lipids with specific chemical structures and physical characteristics can be used to control the mechanical properties of the microbubble shell, such as its elasticity, viscosity, and resistance to rupture.

Several studies have explored the relationship between lipid composition and the mechanical properties of microbubbles. For example, the inclusion of high-molecular-weight lipids, such as distearoylphosphatidylcholine, has been shown to increase the shell elasticity and resistance to rupture [84]. Conversely, the addition of low-molecular-weight lipids, such as phosphatidylethanolamines, can enhance the shell viscosity and damping behavior [84].

Until the time of writing this paper and to the best of the author’s knowledge, no direct method exists to calculate the mechanical properties of microbubbles (MBs) from their lipid composition. While experimental techniques such as atomic force microscopy (AFM), frequency-dependent attenuation, and light scattering provide valuable insights into the mechanical characteristics of MB shells, they require empirical measurements and do not offer a theoretical link between the properties the MB and ELIPS and the mechanical properties of the lipids making them. The development of such a predictive model would represent a significant advancement in the field, enabling more efficient design and the optimization of microbubbles for specific biomedical applications.

### 6.1. Importance of a Composition-Based Predictive Model

The mechanical properties of MBs—such as shell stiffness, elasticity, and permeability—are fundamentally influenced by the composition of the lipid monolayer and bilayer. These properties are critical in determining the stability of MBs under acoustic excitation, their response to ultrasound, and their effectiveness in delivering therapeutic agents. A predictive model that links lipid composition to mechanical behavior would allow researchers to rationally design MBs and ELIPs with specific mechanical properties tailored to the needs of various clinical applications. This would eliminate the need for time-consuming and costly experimental trials, streamlining the development of optimized MB formulations for ultrasound contrast agents and drug delivery systems.

### 6.2. Proposed Approach to Developing the Model

Molecular Dynamics (MD) Simulations: By simulating the behavior of lipid bilayers at the molecular level, it is possible to explore how variations in lipid composition—such as differences in chain length, saturation, and cholesterol content—affect the mechanical properties of MB shells. MD simulations could provide crucial insights into the relationship between molecular structure and macroscopic mechanical properties, serving as the foundation for predictive modeling.

Machine Learning Algorithms: Utilizing machine learning (ML) algorithms trained on existing experimental data of the mechanical properties and feeding it with the composition of the shell lipids parameters (e.g., head group type, tail length, and cholesterol concentration), ML models can predict key mechanical properties such as stiffness, damping, and shell permeability. As more experimental data and MD simulation results become available, these models can be refined to improve their predictive accuracy.

Developing a predictive model that links lipid composition to the mechanical properties of MBs would significantly enhance the design and optimization of these agents for biomedical applications. By combining molecular dynamics simulations, machine learning algorithms, and experimental validation, it is possible to create a robust model that allows for the rational design of MBs with tailored mechanical properties. This would not only streamline the development process but also enable the creation of more effective MBs for use in ultrasound imaging and drug delivery systems.

Computational fluid dynamics (CFD): Several studies have used CFD to simulate microbubble behavior under sonication, offering a significant advancement over traditional lumped-parameter models. Lumped-parameter approaches, such as the Rayleigh–Plesset equation and its common modifications, describe the bubble as a spherically symmetric object whose dynamics are governed by ordinary differential equations (ODEs). These models assume uniform pressure and flow conditions around the bubble, effectively treating the surrounding fluid as an infinite medium and neglecting spatial variations in stress, deformation, or shell interactions. Although it is valuable for capturing basic radial oscillation behaviors and resonance phenomena, lumped models are inherently limited in their ability to simulate non-spherical deformations, translational motion, and interactions with biological boundaries. CFD can incorporate these complications resulting from solid fluid interface by solving Navier–Stokes equations over spatial domains.

Qin and Ferrara (2006) developed an axisymmetric CFD framework that modeled microbubbles oscillations within deformable blood vessels [85], accounting for wall elasticity, fluid viscosity, and acoustic excitation. Their simulations predicted vessel wall stresses and bubble fragmentation thresholds, which aligned with in vitro observations, offering critical insight into ultrasound-mediated drug delivery. The model incorporated real physiological parameters and demonstrated how bubble oscillation intensifies vascular permeability, especially in soft-walled vessels, confirming the relevance of fluid–structure interaction in therapeutic ultrasound applications. Further experimental validation of nonlinear bubble dynamics has been investigated in [86,87] using ultra-high-speed optical imaging to observe subharmonic and nonspherical oscillations of phospholipid-coated MBs in free suspension. These deformation modes, which arise from parametric instabilities, were found to depend sensitively on acoustic pressure and bubble radius and were consistent with predictions from advanced nonlinear models. While full CFD frameworks for echogenic liposomes remain limited, these studies emphasize the importance of incorporating experimental imaging data to verify and refine computational predictions.

## 7. Correlation of Microbubble Mechanical Properties Across Studies

Table 5 summarizes how bubble radius, temperature, and frequency affect the stiffness and friction of microbubbles and echogenic liposomes, based on different measurement techniques. It clearly shows that smaller bubbles consistently have higher stiffness, a result supported by all the experimental methods. This makes sense because smaller bubbles have higher surface tension and shell curvature, making their shells stiffer. However, the effect of bubble size on friction (resistance to movement) is less understood and needs more research, as current data are insufficient.

Temperature generally decreases bubble stiffness, which means that bubbles become softer at higher temperatures. This trend is well-documented for microbubbles, but data on echogenic liposomes (ELIPs) are not consistent and require further investigation. Additionally, frictional properties have been rarely studied concerning temperature, highlighting another important area for future research. Lastly, stiffness clearly increases when measurements are performed at higher frequencies, as demonstrated by acoustic methods. Yet, more studies using direct mechanical tests, such as AFM or optical methods, are needed to confirm these findings and better understand how friction changes with frequency.

**Figure 7:**
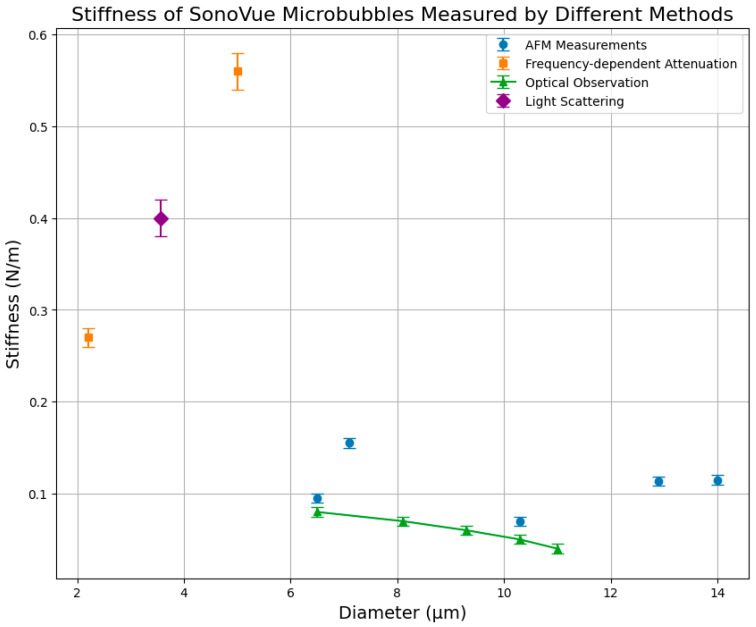
Stiffness of SonoVue microbubbles measured by different experimental methods, including AFM [76], frequency-dependent attenuation [40], optical observation [67], and light scattering [69].

Figure 7 shows the stiffness measurement of different size SonoVue™ MBs measured using several methods. The figure reveals substantial discrepancies across measurement techniques. Frequency-dependent attenuation measurements [40] indicate considerably higher stiffness values compared to optical observation [67], and light scattering [69]. While optical measurements report stiffness values below approximately 0.15 N/m, frequency-dependent attenuation and light scattering methods present notably elevated stiffness values, reaching as high as 0.56 N/m. Such differences can be attributed to methodological variations, including differences in measurement scales, sample handling, bubble populations, and assumptions underlying theoretical models.

## 8. Conclusions

The mechanical properties of microbubbles (MBs) and echogenic liposomes (ELIPs) directly influence their oscillatory behavior, acoustic scattering efficiency, and stability under insonation. These latter characteristics dominates how the acoustic manipulation of the MBs and ELIPs. This review systematically analyzed the primary methodologies used to characterize these properties, identifying key limitations and inconsistencies that hinder accurate cross-comparisons of the properties measured with different methods. Frequency-dependent attenuation measurements are often confounded by nonlinear oscillations, secondary acoustic interactions, and pressure-dependent attenuation artifacts, making results highly system-dependent. Atomic force microscopy (AFM) provides direct stiffness measurements at nanometer resolution but is constrained by substrate effects and single-particle variability, making population-level characterization challenging. Optical observation methods, particularly high-speed imaging, enable real-time analysis of radial dynamic responses but suffer from limited spatial resolution at higher frequencies.

Theoretical models have improved in capturing shell viscoelasticity and lipid monolayer rheology, yet most rely on simplifying assumptions that neglect thermal diffusion, shell permeability, and inter-bubble interactions in dense suspensions. The lack of a standardized reference system in both experimental and computational studies results in significant variability in reported mechanical parameters, such as shell stiffness, friction coefficients, and resonance frequencies. For example, stiffness values for Definity™ microbubbles measured via AFM range from 1.53 ± 0.08 N/m to 3.69 ± 0.76 N/m, depending on excitation pressure and formulation, highlighting inconsistencies in measurement conditions. Similarly, frequency-dependent attenuation studies on SonoVue™ microbubbles report variations in damping coefficients due to differences in insonation pressures and bubble concentrations, affecting shell elasticity estimations.

Despite extensive preclinical use, the clinical translation of microbubbles and echogenic liposomes remains limited due to several practical bottlenecks. One major challenge is the lack of standardization in shell composition, preparation methods, and mechanical characterization protocols, which complicate regulatory approval and batch-to-batch consistency. Stability during storage, reproducibility of gas loading, and polydispersity in particle size also hinder large-scale manufacturing. Furthermore, current models used to predict in vivo behavior are often developed under simplified conditions and may not accurately represent interactions within complex biological environments. Addressing these barriers will require collaborative efforts between researchers, clinicians, and industry stakeholders to develop harmonized testing standards, scalable production workflows, and improved modeling approaches that integrate clinical constraints. Advances in microfluidic synthesis, real-time acoustic monitoring, and AI-assisted data interpretation may provide viable pathways for overcoming these limitations.

To advance the field, future research must focus on harmonizing measurement protocols by defining standard conditions for insonation pressure, temperature control, and lipid formulation. Additionally, computational models should incorporate non-equilibrium thermodynamics, lipid-phase transitions, and shell buckling effects to improve the accuracy of mechanical property predictions. Future studies should also investigate the long-term mechanical stability of ELIPs in physiological conditions, particularly their shell degradation kinetics and gas retention profiles, to optimize formulations for targeted ultrasound-triggered drug release.

## Figures and Tables

**Table 1:** Summary of previously reported shell properties of MBs and ELIPs using frequency-dependent attention measurements.

Study	Agents	f (MHz)	P (kPa)	T (°C)	Method	Dilution	$S_{p}$ (N/m)	$S_{f}\;10^{-6}$ (kg/s)
[33]	Albunex™ (Performed on different size filtered dilutions and single average estimation is calculated.)	0.7–12.5	NR	RT	P/E	1:20,000	8	4
					P/E 12 µm filtered	1:10,000		
					P/E 8 µm filtered	1:10,000		
					P/E 5 µm filtered	1:3500		
					P/E 3 µm filtered	1:1400		
[53]	Definity™	13–29	$P_{pp}=25$	RT	P/E 2 µm filtered	1:15,000	1.51 ± 0.36	0.016 ± 0.016
[53]	Definity™	12–28	$P_{pp}=25$	RT	P/E	1:15,000	1.71 ± 0.24	0.015±0.015
[44]	Definity™	7–15	$P_{pp}=107$	RT	TT	1:15,000	1.64 ± 0.33	0.15 ± 0.02
[44]	Definity™	15–25	$P_{pp}=107$	RT	TT	1:15,000	2.04±0.67	0.01±0.01
[54]	Definity™	2–25	$P_{pp}=30$	25	TT	1:2000	1.76±0.18	0.21±0.07
[54]	Definity™	2–25	$P_{pp}=30$	37	TT	1:2000	1.01±0.07	0.04±0.04
[55]	Definity™	2–25	$P_{-p}=31$ $P_{+p}=24$	25	TT	1:2000	1.76±0.16	0.47±0.05
[55]	Definity™	2–25	$P_{-p}=31$ $P_{+p}=24$	37	TT	1:2000	1.10±0.15	0.20±0.04
[47]	Definity™	2–20	$P_{pp}=11$	25	TT	1:2000	1.53 ± 0.08	1.51 ± 0.12
[47]	Definity™	2–20	$P_{pp}=11$	37	TT	1:2000	1.18 ± 0.16	1.09 ± 0.2
[40]	SonoVue™	0.8–3	$P_{pp}<10$	RT	P/E	1:2000	1.1	0.27
[40]	SonoVue™	3–7	$P_{pp}<10$	RT	P/E (dec 10′)	1:1000	1.1	0.56
[3]	Sonazoid™	1.5–8	-	RT	P/E	-	1.20±0.07	0.48±0.06
[55]	MicroMarker™	2–25	$P_{-p}=31$ $P_{+p}=24$	25	TT	1:200	1.20 ± 0.06	0.62 ± 0.03
[55]	MicroMarker™	2–25	$P_{-p}=31$ $P_{+p}=24$	37	TT	1:200	1.90 ± 0.05	0.87 ± 0.02
[55]	EggPC:DPPE: DPPG:CH (69:8:8:15)-(Air)	2–25	$P_{-p}=31$ $P_{+p}=24$	25	TT	1:200	1.13 ± 0.13	0.82 ± 0.04
[55]	EggPC:DPPE: DPPG:CH (69:8:8:15)-(Air)	2–25	$P_{-p}=31$ $P_{+p}=24$	37	TT	1:200	1.49 ± 0.20	1.41 ± 0.07
[55]	EggPC:DPPC:DPPE:DPPG:CH (27:42:8:8:15)-(Air)	2–25	$P_{-p}=31$ $P_{+p}=24$	25	TT	1:200	1.98 ± 0.10	0.41 ± 0.03
[55]	EggPC:DPPC:DPPE:DPPG:CH (27:42:8:8:15)-(Air)	2–25	$P_{-p}=31$ $P_{+p}=24$	37	TT	1:200	3.10 ± 0.25	1.01 ± 0.07
[55]	DPPC:DOPC:DPPG:CH (46:24:24:6)-(Air)	2–25	$P_{-p}=31$ $P_{+p}=24$	25	TT	1:200	3.69 ± 0.76	1.88 ± 0.23
[55]	DPPC:DOPC:DPPG:CH (46:24:24:6)-(Air)	2–25	$P_{-p}=31$ $P_{+p}=24$	25	TT	1:200	5.16 ± 0.37	2.09 ± 0.10
[47]	DPPC:DSPE-PEG2000 (94:6)-(C_3_F_8_)	2–20	$P_{pp}=11$	25	TT	1:20	0.11 ± 0.02	0.31 ± 0.03
[47]	DPPC:DSPE-PEG2000 (94:6)-(C_3_F_8_)	2–20	$P_{pp}=11$	37	TT	1:20	0.15 ± 0.01	0.29 ± 0.01

Here, f is the frequency, P is the pressure, $P_{pp}$ is peak-to-peak pressure, $P_{-p}$ is peak negative pressure, $P_{+p}$ is peak positive pressure, T is the temperature, $S_{p}$ is the shell stiffness, and $S_{f}$ is the shell friction. RT is not-specified room temperature, P/E is pulse echo, NR is not reported, TT is through transmission, and P/E is pulse echo.

**Table 2:** Dilation viscosity and interfacial tension of specific agents calculated using Sarkar’s model.

MBs	$\kappa^{s}$ (ms Pa)	γ (N/m)
Albunex™	0.05	0.78
Optison™	0.08	0.9
Quantison™	4.24	38.34
Sonazoid™	0.01	0.6

**Table 4:** Summary of microbubble shell stiffness measurements obtained using light scattering methods.

Study	Agents	P (kPa)	f (MHz)	Radius (µm)	Model	Elasticity	Viscosity
[71]	Optison™	340	1.8	1.5	Morgan et al.	Assumed χ = 0 N/m	$\epsilon \eta_{s}$ = 6 nm Pa s
	Sonazoid™	390	1.8	1.1	Morgan et al.		$\epsilon \eta_{s}$ = 2 nm Pa s
[69]	SonoVue™	150	2.5	1.78	Marmottant	χ = 0.3 N/m	$k_{s}\;=\;3.2\times 10^{-9}\;kg/s$
					Sarkar	$\sigma_{i}\;=\;0.4\;N/m$	$k_{s}\;=\;4\times 10^{-9}\;kg/s$
					Hoff	$G_{s}\;=\;20\;MPa$	$\eta_{s}\;=\;0.6\;Pa\;s$
[72]	Definity™	308	1	1.18	Marmottant	χ = 0.5 N/m	$k_{s}\;=\;2.8\times 10^{-9}\;kg/s$

χ is the shell elasticity, $k_{s}$ is the dilation viscosity, $\sigma_{i}$ is the interfacial tension, $G_{s}$ is the shear modulus, $\eta_{s}$ is the shear viscosity, and ϵ is the shell thickness.

**Table 5:** Correlations between microbubble mechanical properties (stiffness and friction) and factors like radius, temperature, and frequency, as confirmed by different experimental methods.

Influencing Factor	Property	Frequency-Dependent Attenuation	Optical Observation	AFM	Light Scattering
Radius	Stiffness	Confirmed inverse relationship [40,44,77,82]	Confirmed inverse relationship [62,63,64]	Confirmed inverse relationship [66,73,75,76]	Confirmed inverse relationship [69]
Radius	Friction	Weakly explored; limited evidence [44,68,70]	Not extensively explored	Not extensively explored	Not extensively explored
Temperature	Stiffness	Confirmed inverse relationship [44,55]	Limited exploration, some inconsistency [62,63,80]	Not extensively explored	Not extensively explored
Temperature	Friction	Weak/inconclusive [44,68,70]	Not extensively explored	Not extensively explored	Not extensively explored
Frequency	Stiffness	Frequency-dependent increase clearly observed [40,44,77,82]	Not extensively explored	Not extensively explored	Not extensively explored
Frequency	Friction	Frequency-dependent increase observed [40,44,68,70]	Not extensively explored	Not extensively explored	Not extensively explored

The green background indicates relationships extensively confirmed, gray background denotes relationships that have been explored with limited or inconsistent findings, and orange background highlights relationships that have not been extensively explored or lack sufficient evidence.
